# Time Delay Complex Chen Chaotic System and Secure Communication Scheme for Wireless Body Area Networks

**DOI:** 10.3390/e22121420

**Published:** 2020-12-16

**Authors:** Fangfang Zhang, Sen Leng, Zhengfeng Li, Cuimei Jiang

**Affiliations:** 1 School of Electrical Engineering and Automation, Qilu University of Technology (Shandong Academy of Sciences), Jinan 250353, China; 1043118083@stu.qlu.edu.cn (S.L.); 1043119266@stu.qlu.edu.cn (Z.L.); 2 Shandong Computer Science Center (National Supercomputer Center in Jinan), Shandong Artificial Intelligence Institute, Qilu University of Technology (Shandong Academy of Sciences), Jinan 250101, China; 3 School of Mathematics and Statistics, Qilu University of Technology (Shandong Academy of Sciences), Jinan 250353, China; jcm2017@qlu.edu.cn

**Keywords:** complex Chen system, time delay, controller, secure communication scheme

## Abstract

Although many chaotic systems with time delays have been studied in recent years, most studies have only focused on the theoretical level, without special applications. Therefore, we present a basic introduction of a time delay complex Chen chaotic system, including the influence of parameter changes and time delay factors on the time delay system. On the basis of complex modified projection synchronization (CMPS), we detail the design of a new controller and communication scheme and apply this communication scheme to a wireless body area network (WBAN), in order to encrypt and decrypt body data collected by sensors. Finally, we perform a numerical simulation, demonstrating the effectiveness of the proposed communication scheme.

## 1. Introduction

In 1977, Mackey and Glass [1], while studying first-order non-linear delay differential equations to describe physical control systems, first discovered that chaos exists in delay systems. Following this, researchers began to pay close attention to time delay chaotic systems, finding that those with a time delay can better describe real physical processes than systems without a time delay. On the basis of the stability of a class of linear-system theory, the synchronization of Chen systems with time-varying delay has been discussed. Research on time delay chaotic systems with real variables, as well as on time delay chaotic systems with complex variables, has made great progress. Since Fowler et al. [2] introduced the complex Lorenz system, research on the characteristics of complex chaotic systems and their synchronization has attracted much attention [3,4,5,6,7,8]. However, time delay is an inevitable factor in actual complex chaotic systems. Therefore, many scholars have studied complex chaotic systems with time delay [9,10,11].

Since Leon O. Chua realized chaotic synchronization by circuits [12], the application of chaotic systems in secure communication has become a hot issue in the field of information security. Similarly to random signals, chaotic signals have very complex trajectories, are difficult to predict, and have inherent concealment, which make them suitable as carriers for secure communication [13,14,15,16,17]. Generally speaking, the information is encrypted as streams with chaotic characteristics at the sending end. At the receiving end, the correct information is decrypted from the received signal. Chaotic secure communication requires the synchronization of chaotic systems at the sending and receiving ends. A chaotic system is completely determined by a non-linear system of equations, including its parameters and initial conditions. The most interesting fact is that a determinate system can produce a large number of complex, uncorrelated, and quasi-random chaotic sequences. Time delay is inevitable in nature, where time delay systems have more complicated dynamic characteristics than ordinary chaotic systems. Therefore, time delay complex chaotic systems have become a prominent topic in current chaotic secure communication research.

Countless scholars have conducted research on secure communication, in order to achieve high security levels. In order to achieve fast communication, Chee and Xu used the proportional characteristics of projection synchronization (PS) and extended binary to M-ary (M>2) systems. Mahmoud G.M. [18,19] studied the projection synchronization of complex hyperchaotic systems and proposed corresponding communication schemes. As the rapid communication of projection synchronization and the unpredictability of the scale factor can increase communication security, PS has been widely used. On the basis of the definition of real scale-factor projection synchronization and modified projection synchronization (MPS) [20,21], the definition of modified projection synchronization with a complex scale factor—in fact, complex modified projection synchronization (CMPS)—has been proposed by Zhang et al. [22] and Mahmoud [23], almost at the same time. If the real part of a complex scale factor is 1 and its imaginary part is 0, it is complete synchronization (CS); if the real part of the complex scale factor is −1 and the imaginary part is 0, it is the anti synchronization (AS); if the real part of the complex scale factor is a real number and the imaginary part is 0, it is the projection synchronization (PS) of the real scale factor; if only the imaginary part is 0, it is the modified projection synchronization (MPS) of the real scale factor; if the complex scale factor is *j*, it is complex complete synchronization (CCS). Therefore, CS, AS, PS, MPS, and CCS are all special cases of CMPS. As complex numbers have real and imaginary parts, a complex scale factor can be more arbitrary and unpredictable than a real scale factor, such that the calculation of complex numbers is more complicated than that of real numbers and, so, it is more difficult to obtain signal information from transmitted information. Moreover, CMPS establishes a connection between real and complex chaos, thus increasing the selection range of the chaos generator at the sending and receiving ends. Studying CMPS can increase the complexity of synchronization and, consequently, the diversity and security of communications.

Recently, great improvements in integrated chips and wireless communications have promoted the development of wireless body area networks (WBANs). The security of data transmission in WBANs is more important and essential. Due to the low cost and high-security properties of chaotic signals, the implementation of chaos communication in WBANs is an effective and promising solution, which can improve communication security in WBANs. However, to the best of our knowledge, secure communication in WBANs based on the CMPS of complex chaos under noisy conditions has seldom been studied in the literature. The main contributions of this paper are as follows:

(1) A basic introduction of the time delay complex Chen system, including the influence of parameter changes and time delay factors on the time delay system, is presented.

(2) On the basis of complex modified projection synchronization (CMPS), a new controller and communication scheme are designed.

(3) This communication scheme is applied to a wireless body area network (WBAN) for encrypting and decrypting body data that are collected by sensors.

The rest of this article is organized as follows: In Section 2, the time delay characteristics of the complex Chen system are analyzed and its randomness, symmetry, and unpredictability are discussed. In Section 3, the innovation and advantages of the communication scheme for wireless body area networks are introduced. In Section 4, the CMPS controller design is described. In Section 5, the feasibility of the scheme is verified through MATLAB simulation. In Section 6, the security of the communication scheme for WBAN is analyzed. Section 7 summarizes this article.

## 2. Characteristics of Time Delay Complex Chen System

Consider the following time delay complex Chen system:

(1) $$\begin{array}{c} \begin{array}{c} \left\{\begin{array}{ccc} {\dot{y}}_{1} & = & a_{1}\left(y_{2}-y_{1}\right),\\ {\dot{y}}_{2} & = & \left(a_{2}-a_{1}\right)y_{1}+a_{2}y_{2}-y_{1}y_{3}\left(t-\tau \right),\\ {\dot{y}}_{3} & = & -a_{3}y_{3}\left(t-\tau \right)+\left(1/2\right)\left({\bar{y}}_{1}y_{2}+y_{1}{\bar{y}}_{2}\right), \end{array}\right. \end{array} \end{array}$$

where $0\leq \tau \leq \tau_{m}$ is the time delay factor, $y_{1}=u_{1}+ju_{2}$ and $y_{2}=u_{3}+ju_{4}$ are the complex state variables, and $y_{3}=u_{5}$ is the real state variable. Overbar ${\bar{y}}_{1}\left({\bar{y}}_{2}\right)$ stands for the complex conjugate of $y_{1}\left(y_{2}\right)$, and ${\left(a_{1},\;a_{2},\;a_{3}\right)}^{T}$ is the real parameter vector.

Separating the real and imaginary parts of each variable in System (1), we obtain

(2) $$\begin{array}{c} \begin{array}{c} \left\{\begin{array}{ccc} {\dot{u}}_{1} & = & a_{1}\left(u_{3}-u_{1}\right),\\ {\dot{u}}_{2} & = & a_{1}\left(u_{4}-u_{2}\right),\\ {\dot{u}}_{3} & = & \left(a_{2}-a_{1}\right)u_{1}-u_{1}u_{5}\left(t-\tau \right)+a_{2}u_{3},\\ {\dot{u}}_{4} & = & \left(a_{2}-a_{1}\right)u_{2}-u_{2}u_{5}\left(t-\tau \right)+a_{2}u_{4},\\ {\dot{u}}_{5} & = & -a_{3}u_{5}\left(t-\tau \right)+\left(u_{1}u_{3}+u_{2}u_{4}\right). \end{array}\right. \end{array} \end{array}$$

### 2.1. Chaos Attractor

**Figure 1:**
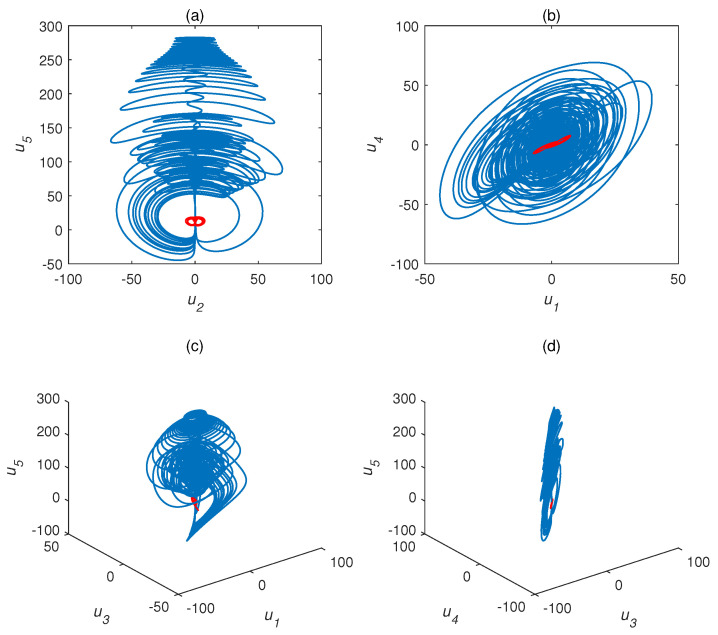
Chaotic attractor projections for time delay complex Chen system ($a_{1}=35,\;a_{2}=23,\;a_{3}=1$). Blue attractor, complex Chen system with τ=1 s; red attractor, complex Chen system with τ=0 s). (**a**) $u_{2}-u_{5}$, (**b**) $u_{1}-u_{4}$, (**c**) $u_{1}-u_{3}-u_{5}$, and (**d**) $u_{3}-u_{4}-u_{5}$.

**Figure 2:**
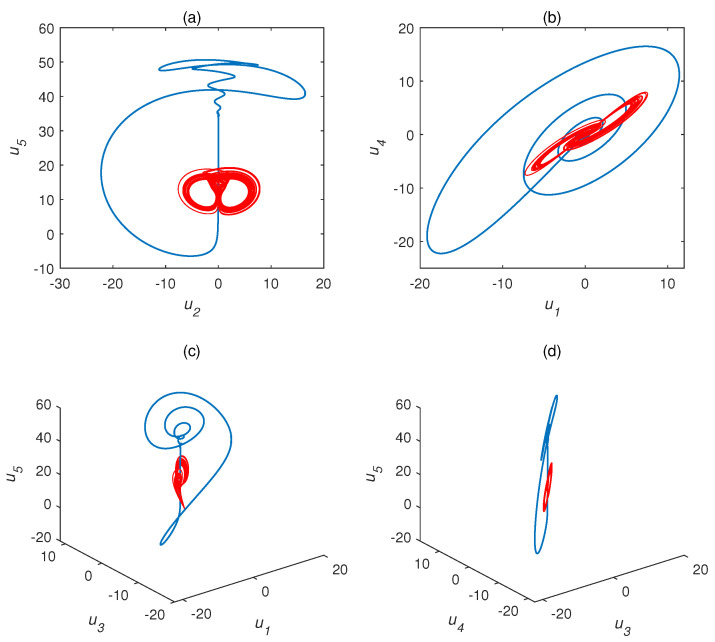
Chaotic attractor projections for time delay complex Chen system ($a_{1}=35,\;a_{2}=23,\;a_{3}=1$). Blue attractor, complex Chen system with τ=1.5 s; red attractor, complex Chen system with τ=0 s. (**a**) $u_{1}$, (**b**) $u_{2}$, (**c**) $u_{3}$, (**d**) $u_{4}$.

We adopted $a_{1}=35,\;a_{2}=23,\;a_{3}=1$, and ${\left(1,2,3,4,1\right)}^{T}$ as the initial conditions. Their different chaotic attractor projections are shown as blue attractors in Figure 1 (τ=1 s) and Figure 2 (τ=1.5 s), where the red attractor is a complex Chen system with τ=0 s. The projection of the time delay complex Chen system is quite different than the original system. The attractor of the former occupies much more space than the latter. Time delay systems produce time-series data with extremely high randomness and unpredictability. The time delay complex Chen system with τ=1 s was chaotic, while the Chen system with τ=1.5 s exhibited a limit cycle. This indicates that, under the same parameters and initial values, different time delay factors cause hugely different outputs.

### 2.2. Symmetry and Initial Value Sensitivity

System (2) was unchanged after introducing the following transformation: $\left(u_{1},\;u_{2},\;u_{3},\;u_{4},\;u_{5}\right)\to \left(-u_{1},\;-u_{2},\;-u_{3},\;-u_{4},\;u_{5}\right)$; thus, the system exhibited symmetry about the $u_{5}$ axis. This symmetry held for all parameters; namely, $a_{1}$, $a_{2}$, and $a_{3}$.

**Figure 3:**
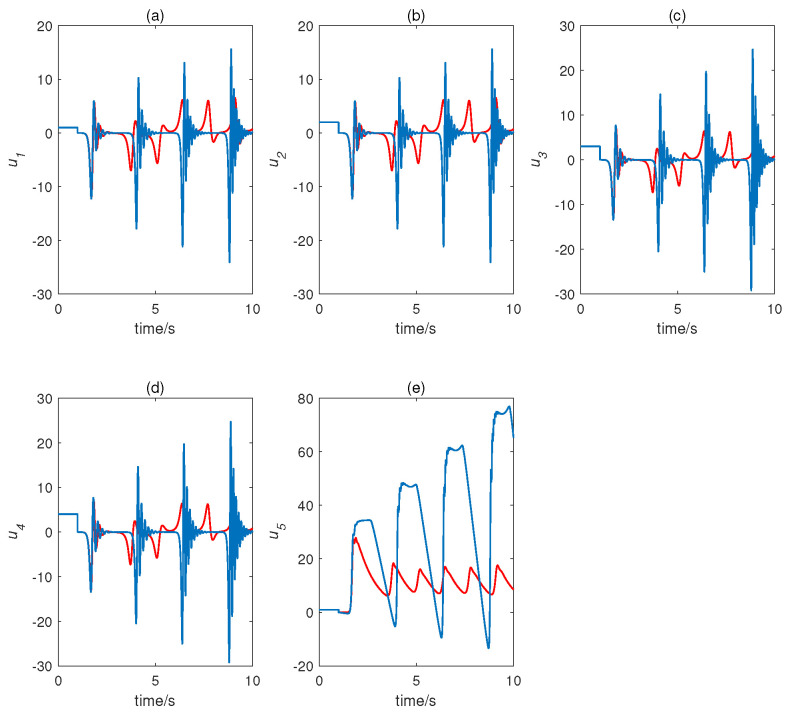
Initial sensitivity of time delay complex Chen system. Red curve represents initial state ${\left(1,2,3,4,1\right)}^{T}$; blue curve represents initial state ${\left(1,2,3,4,1.001\right)}^{T}$). (**a**) $u_{1}$, (**b**) $u_{2}$, (**c**) $u_{3}$, (**d**) $u_{4}$, and (**e**) $u_{5}$.

When τ=2 s, we selected two close initial conditions—namely, ${\left(1,2,3,4,1\right)}^{T}$ and ${\left(1,2,3,4,1.001\right)}^{T}$—and obtained the evolution of states, as shown in Figure 3. The time delay complex Chen system displayed sensitive dependence on the initial conditions.

### 2.3. Lyapunov Exponent and Bifurcation Diagram

**Figure 4:**
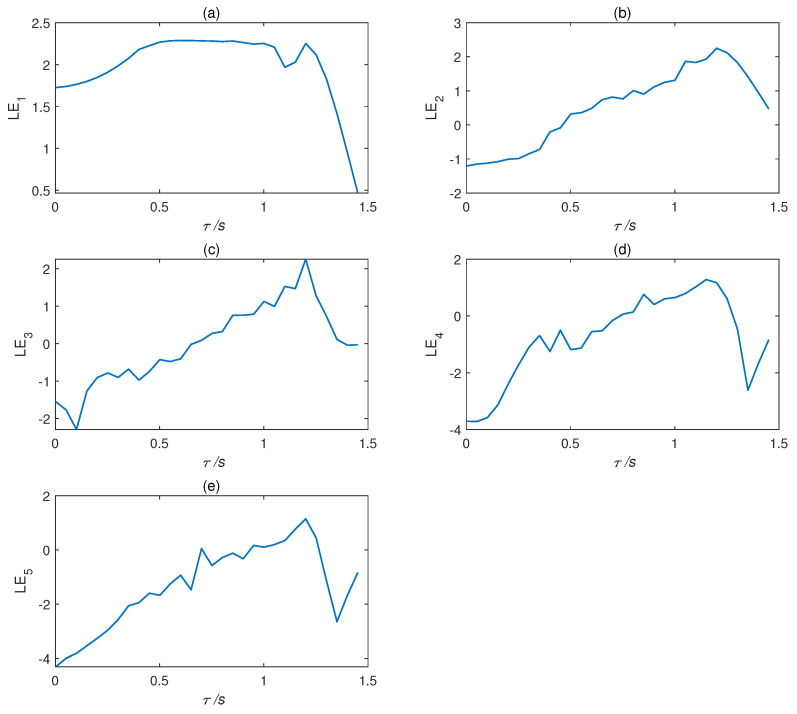
Lyapunov exponent curves of time delay complex Chen system: (**a**) $LE_{1}$, (**b**) $LE_{2}$, (**c**) $LE_{3}$, (**d**) $LE_{4}$, and (**e**) $LE_{5}$.

The Lyapunov exponent can quantitatively reflect the chaotic performance of a system. Letting $a_{1}=35$, $a_{2}=23$, and $a_{3}=1$, we obtained the Lyapunov exponent curve of the time delay complex Chen system, as shown in Figure 4. Table 1 shows some values of the Lyapunov exponent. Obviously, the characteristics of the time delay complex Chen system and the number of positive Lyapunov exponents are related to the time delay factor τ, which also indicates the randomness and unpredictability of the delay system.

**Figure 5:**
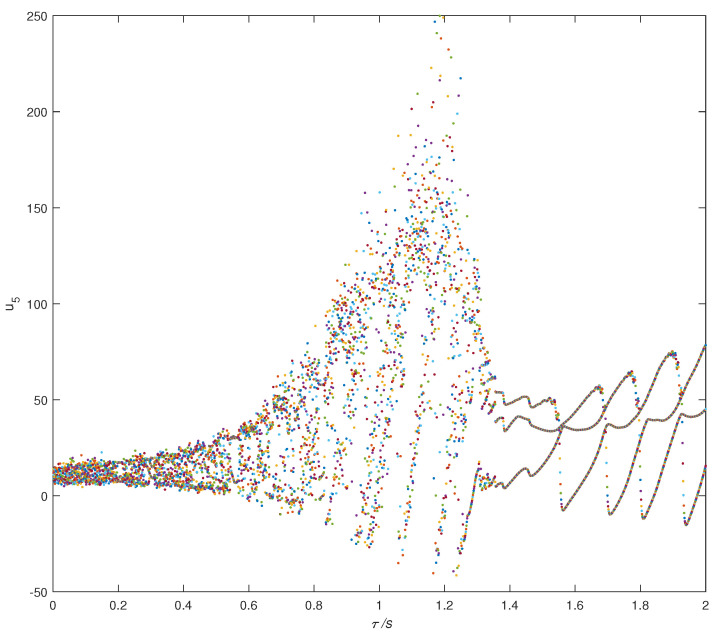
Bifurcation diagram of time delay complex Chen system.

In order to more intuitively see the change of system characteristics with time delay factors, the bifurcation diagram of the system output $u_{5}$ and τ was produced, as shown in Figure 5. When 0.5<τ≤1.3, the system was chaotic; when τ=1.5, the system gradually exhibited limit-cycle behavior. Therefore, by choosing different time delay parameters, τ, the time delay complex system produced different dynamic phenomena. The existence of time delay parameters increased the complexity of the Chen system and, thus, the security of confidential communication. The design of a secure communication scheme based on the CMPS of the time delay complex Chen system is described in the following section.

## 3. Communication Scheme Based on CMPS

### 3.1. Design of CMPS Controller

Consider the following *n*-dimensional complex chaotic non-linear system as a response system:

(3) $$\begin{array}{c} \dot{x}=f\left(x\right)+v+\epsilon_{1}, \end{array}$$

where $x={\left(x_{1},x_{2},\ldots ,x_{n}\right)}^{T}$ is the complex state vector, $x=x^{r}+jx^{i}$, and the superscripts *r* and *i* denote the real and imaginary parts of the complex state vector, respectively. Let $x_{1}=x_{1}^{r}+jx_{1}^{i},x_{2}=x_{2}^{r}+jx_{2}^{i},\ldots ,x_{n}=x_{n}^{r}+jx_{n}^{i}$; then, $x^{r}={\left(x_{1}^{r},x_{2}^{r},\ldots ,x_{n}^{r}\right)}^{T},x^{i}={\left(x_{1}^{i},x_{2}^{i},\ldots ,x_{n}^{i}\right)}^{T}$. f(x) is an n×m complex matrix, with its elements being functions of complex state variables. $f={\left(f_{1},f_{2},\ldots ,f_{n}\right)}^{T}$ is a non-linear complex function vector. The controller to be designed is $v=v^{r}+jv^{i}$, where $v^{r}={\left(v_{1}^{r},v_{2}^{r},\ldots ,v_{n}^{r}\right)}^{T}$ and $v^{i}={\left(v_{1}^{i},v_{2}^{i},\ldots ,v_{n}^{i}\right)}^{T}$. $\epsilon_{1}={\left(\epsilon_{11},\epsilon_{12},\ldots ,\epsilon_{1n}\right)}^{T}$ indicates external bounded interference, such that $|\epsilon_{1l}|<\rho \left(l=1,2,\ldots ,n\right)$, where $\rho_{1}$ is a positive constant (|·| is the modulus of a complex number).

Consider the drive chaotic complex system $y={\left(y_{1},y_{2},\ldots ,y_{n}\right)}^{T}$ satisfying

(4) $$\begin{array}{c} \dot{y}=g\left(y\right)+\epsilon_{2}, \end{array}$$

where $y={\left(y_{1},y_{2},\ldots ,y_{n}\right)}^{T}$ is the complex state vector, $y=y^{r}+jy^{i}$, with $y^{r}={\left(y_{1}^{r},y_{2}^{r},\ldots ,y_{n}^{r}\right)}^{T}$ and $y^{i}={\left(y_{1}^{i},y_{2}^{i},\ldots ,y_{n}^{i}\right)}^{T}$. $g={\left(g_{1},g_{2},\ldots ,g_{n}\right)}^{T}$ is a non-linear complex function vector. $\epsilon_{2}={\left(\epsilon_{21},\epsilon_{22},\ldots ,\epsilon_{2n}\right)}^{T}$ is bounded external interference, such that $|\epsilon_{2l}|<\rho_{2}\left(l=1,2,\ldots ,n\right)$, where $\rho_{2}$ is a positive constant.

For the response System (3) and drive System (4), if there is a complex constant matrix $H=diag\left\{h_{1},h_{2},\ldots ,h_{n}\right\}=diag\left\{h_{1}^{r}+jh_{1}^{i},h_{2}^{r}+jh_{2}^{i},\ldots ,h_{n}^{r}+jh_{n}^{i}\right\}$ satisfying

(5) $$\begin{array}{c} \begin{array}{cc} \lim_{t\to \infty }{∥e\left(t\right)∥}^{2} & =\lim_{t\to \infty }{∥x\left(t\right)-Hy\left(t\right)∥}^{2}\\ & =\lim_{t\to \infty }\left(∥x^{r}\left(t\right)-H^{r}y^{r}\left(t\right)+H^{i}y^{i}\left(t\right)\right){∥}^{2}\\ & \;\;\;\;+∥x^{i}\left(t\right)-H^{r}y^{i}\left(t\right)-H^{i}y^{r}\left(t\right){∥}^{2})\\ & =0, \end{array} \end{array}$$

where e(t) is an error vector, then the chaotic Systems (3) and (4) realize complex modified projection synchronization.

Therefore, on the basis of active control and [22,24,25], we designed the controller as

(6) $$\begin{array}{c} v=Hg(y)-f(x)+Ke, \end{array}$$

where $K=diag(k_{1},k_{2},\ldots ,k_{n})$ is the real control intensity matrix. The specific proof process can be found in [22,24].

### 3.2. Communication Scheme for Wireless Body Area Network

**Figure 6:**
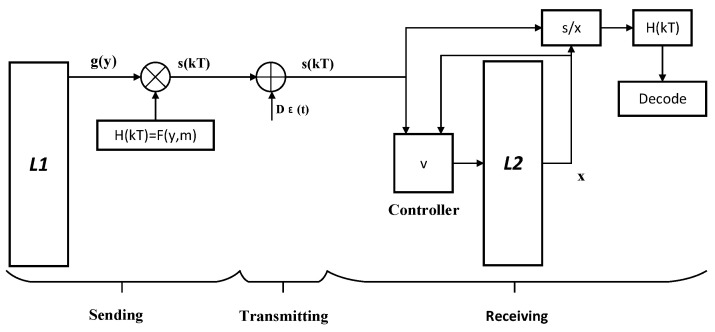
Communication scheme based on complex modified projection synchronization (CMPS) for a wireless body area network.

In wireless body area networks, sensors are used to measure the temperature, blood pressure, heart rate, and other physiological information of users, first transmitting the measurement results to smart mobile devices (e.g., smartphones) and, then, sending them to a telematics terminal. After mutual authentication between the smart mobile device and the telematics terminal, the smart mobile device encrypts the collected sensor information (clear text) and the shared chaotic signal (produced from time delay complex Chen system with the same parameters and initial values) of the telematics terminal after authentication and sends them to storage devices for remote services. After the remote service storage device successfully receives the data, it synchronously decrypts the data, according to the complex modified proportional projection, such that the plain-text is obtained in the data buffer of the remote service storage device. The communication block diagram is based on CMPS, as shown in Figure 6.

We use L1 and L2 to represent smart devices (transmitting end) and the telematics terminal (receiving end), respectively. The plain-text signals to be transmitted are body temperature $h_{1}^{r}$, high blood pressure value $h_{1}^{i}$, low blood pressure value $h_{2}^{r}$, heart rate $h_{2}^{i}$, and blood sugar $h_{3}$, which are all complex scale factors in CMPS; the duration of every sample was 1 min. Then, the ciphertext transmission signal

(7) $$\begin{array}{c} \begin{array}{cc} s(t) & =Hg(y)+D\varepsilon (t)\\ & =diag\left\{h_{1}^{r}+jh_{1}^{i},h_{2}^{r}+jh_{2}^{i},h_{3}\right\}g\left(y\right)\\ & +diag\{D\varepsilon (t)+jD\varepsilon (t),D\varepsilon (t)+jD\varepsilon (t),D\varepsilon (t)\} \end{array} \end{array}$$

is expanded into five channels; that is,

(8) $$\begin{array}{c} \left\{\begin{array}{cc} & s_{1}^{r}\left(t\right)=h_{1}^{r}g_{1}{\left(y\right)}^{r}-h_{1}^{i}g_{1}{\left(y\right)}^{i}+D\varepsilon \left(t\right),\\ & s_{1}^{i}\left(t\right)=h_{1}^{r}g_{1}{\left(y\right)}^{i}+h_{1}^{i}g_{1}{\left(y\right)}^{r}+D\varepsilon \left(t\right),\\ & s_{2}^{r}\left(t\right)=h_{2}^{r}g_{2}{\left(y\right)}^{r}-h_{2}^{i}g_{2}{\left(y\right)}^{i}+D\varepsilon \left(t\right),\\ & s_{2}^{i}\left(t\right)=h_{2}^{i}g_{2}{\left(y\right)}^{r}+h_{2}^{r}g_{2}{\left(y\right)}^{i}+D\varepsilon \left(t\right),\\ & s_{3}\left(t\right)=h_{3}g_{3}\left(y\right)+D\varepsilon \left(t\right), \end{array}\right. \end{array}$$

where Dε(t) simulates the noise generated by the communication channel and noise source. The controller *v* at the receiving end was designed as (6), which contains the transmission signal s(t) and v=s(t)−f(x)+ke. As CMPS occurs, x(t) approaches H(t)y(t) ($H\left(t\right)=diag\left\{h_{1}^{r}+jh_{1}^{i},h_{2}^{r}+jh_{2}^{i},h_{3}\right\}$). The information signal recovered at the receiving end is $H_{g}\left(t\right)=diag\left\{h_{g1},h_{g2},h_{g3}\right\}=diag\left\{x_{1}/y_{1},x_{2}/y_{2},x_{3}/y_{3}\right\}$.

Compared with other examples of communication systems [26,27,28,29,30,31,32,33], the CMPS-based communication scheme has the following advantages:

(1) As shown in Equation (7), two layers masked by noise and chaotic signals are used here, where the chaotic signal is the derivative of the system state variable, not the state variable itself, which differs from traditional chaotic masking, thus increasing the difficulty of decoding.

(2) The method of recovering plain-text signals is essentially different. In traditional chaos masking, a transmission signal is used to subtract a synchronized chaotic signal, in order to recover a plain-text signal. The effect of channel noise is theoretically ignored and the bit error rate (BER) cannot be guaranteed to be zero. In our communication scheme, we use a CMPS controller to ensure that the signal at the receiving end is equal to the product of the plain-text and chaotic signals. Therefore, BER = 0 for the recovered signal in theory.

(3) Compared with CS, which is often used for chaotic communication, CMPS can be observed from partially or completely different dynamic systems. CS requires the sender and receiver to be identical, which is difficult in practical applications; especially for long-term device operation. With regard to CMPS, the channel transmitter and receiver can be the same or different, which avoids this problem at a basic level.

(4) The transmission signal involves complex operations, such as the multiplication of complex numbers and taking derivatives. The ciphertext signal transmitted by each channel is a combination of five pieces of plain-text information and chaotic signal derivatives. The smartphone samples the sensor every minute. Even if a certain channel is intercepted, it is extremely hard to crack the plain-text in one minute, thereby increasing attack resistance and information security.

(5) The dynamic characteristics of the time delay complex Chen system are more complicated than those of the time delay-free complex system, which can increase the secrecy of chaotic communication.

## 4. Controller Design

We adopted the following time delay complex Chen system as the drive system L1 at the sending end and response system L2 at the receiving end:

(9) $$\begin{array}{c} L1:\begin{array}{c} \left\{\begin{array}{ccc} {\dot{y}}_{1} & = & a_{1}\left(y_{2}-y_{1}\right),\\ {\dot{y}}_{2} & = & \left(a_{2}-a_{1}\right)y_{1}+a_{2}y_{2}-y_{1}y_{3}\left(t-\tau \right),\\ {\dot{y}}_{3} & = & -a_{3}y_{3}\left(t-\tau \right)+\left(1/2\right)\left({\bar{y}}_{1}y_{2}+y_{1}{\bar{y}}_{2}\right), \end{array}\right. \end{array} \end{array}$$

(10) $$\begin{array}{c} L2:\begin{array}{c} \left\{\begin{array}{ccc} {\dot{x}}_{1} & = & a_{1}\left(x_{2}-x_{1}\right)+v_{1}+jv_{2},\\ {\dot{x}}_{2} & = & \left(a_{2}-a_{1}\right)x_{1}+a_{2}x_{2}-x_{1}x_{3}\left(t-\tau \right)+v_{3}+jv_{4},\\ {\dot{x}}_{3} & = & -a_{3}x_{3}\left(t-\tau \right)+\left(1/2\right)\left({\bar{x}}_{1}x_{2}+y_{1}{\bar{x}}_{2}\right)+v_{5}, \end{array}\right. \end{array} \end{array}$$

where $y_{1}=u_{1}+ju_{2}$ and $y_{2}=u_{3}+ju_{4}$ are the complex state variables of the drive system L1, $x_{1}=u_{1}^{\prime }+ju_{2}^{\prime }$, and $x_{2}=u_{3}^{\prime }+ju_{4}^{\prime }$ are the complex state variables of the response system L2, and the real variables are $y_{3}=u_{5}$ and $x_{3}=u_{5}^{\prime }$. The controller is $v=diag\{v_{1}+jv_{2},v_{3}+jv_{4},v_{5}\}$. Here, τ=1 s and the system is chaotic, as shown as Figure 1.

The system parameters ($a_{1}=35,\;a_{2}=23,\;a_{3}=1$, τ=1 s) and initial conditions ($u\left(0\right)={\left(1,2,3,4,1\right)}^{T}$, $u^{\prime }\left(0\right)={\left(1,2,3,4,1\right)}^{T}$) are certified by the sender and receiver. We employed controller (6), with $k_{1}=k_{2}=k_{3}=k_{4}=k_{5}=-100$. Therefore, the controller was designed as:

(11) $$\begin{array}{c} \begin{array}{c} \left\{\begin{array}{cc} v_{1}^{r} & =s_{1}^{r}-f_{1}^{r}\left(x\right)+k_{1}e_{1}^{r}\\ & =s_{1}-a_{1}\left(u_{3}^{\prime }\left(t-\tau \right)-u_{1}^{\prime }\right)-k_{1}\left(u_{1}^{\prime }-h_{1}*u_{1}+h_{2}*u_{2}\right)\\ v_{1}^{i} & =s_{1}^{i}-f_{1}^{i}\left(x\right)+k_{2}e_{1}^{i}\\ & =s_{2}-a_{1}\left(u_{4}^{\prime }-u_{2}^{\prime }\right)-k_{2}\left(u_{2}^{\prime }-h_{1}*u_{2}-h_{2}*u_{1}\right)\\ v_{2}^{r} & =s_{2}^{r}-f_{2}^{r}\left(x\right)+k_{3}e_{2}^{r}\\ & =s_{3}-\left(a_{2}-a_{1}\right)u_{1}^{\prime }-a_{2}u_{3}^{\prime }\left(t-\tau \right)-u_{1}^{\prime }u_{5}^{\prime }\\ & -k_{3}\left(u_{3}^{\prime }\left(t-\tau \right)-h_{3}*u_{3}+h_{4}*u_{4}\right)\\ v_{2}^{i} & =s_{2}^{i}-f_{2}^{i}\left(x\right)+k_{4}e_{2}^{i}\\ & =s_{4}-\left(a_{2}-a_{1}\right)u_{2}^{\prime }-a_{2}u_{4}^{\prime }-u_{2}^{\prime }u_{5}^{\prime }\\ & -k_{4}\left(u_{4}^{\prime }-h_{3}*u_{4}-h_{3}*u_{4}\right)\\ v_{3} & =s_{3}-f_{3}\left(x\right)+k_{5}e_{5}\\ & =s_{5}+a_{3}u_{3}^{\prime }\left(t-\tau \right)-\left(u^{\prime }1*u_{3}^{\prime }\left(t-\tau \right)+u_{2}^{\prime }*u_{4}^{\prime }\right)\\ & -k_{5}\left(u_{5}^{\prime }-h_{5}*u_{5}\right) \end{array}\right. \end{array} \end{array}$$

**Figure 7:**
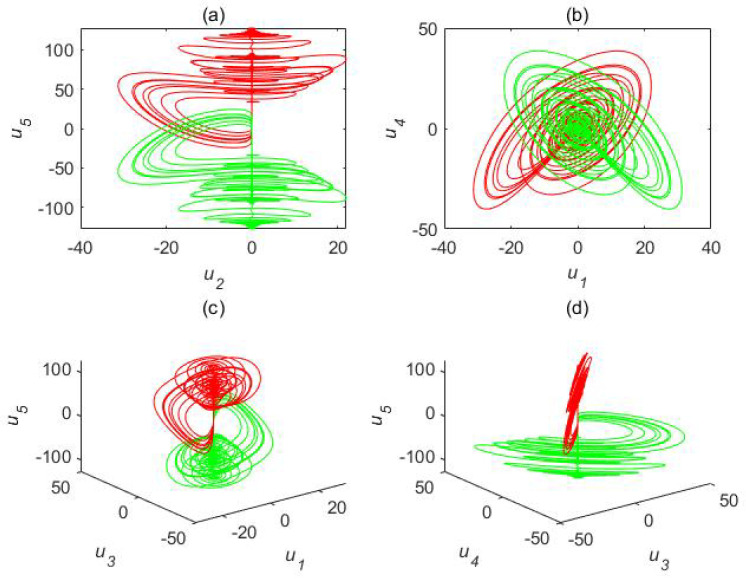
Attractor projections of special synchronization for time delay complex Chen system. Red attractor, system L1; green attractor, system L2. (**a**) $u_{2}-u_{5}$, (**b**) $u_{1}-u_{4}$, (**c**) $u_{1}-u_{3}-u_{5}$, and (**d**) $u_{3}-u_{4}-u_{5}$.

First, we chose $h_{1}=h_{2}=j,h_{3}=-1$, which means that $e_{1}=\left(u_{1}^{\prime }+u_{2}\right)+j\left(u_{2}^{\prime }-u_{1}\right)=0$, $e_{2}=\left(u_{3}^{\prime }\left(t-\tau \right)+u_{4}\right)+j\left(u_{4}^{\prime }-u_{3}\right)=0$, and $e_{3}=u_{5}^{\prime }+u_{5}=0$. In fact, $x_{1},x_{2}$ and $y_{1},y_{2}$ realize CCS, while $x_{3}$ and $y_{3}$ realize anti-synchronization. The attractor projections of this synchronization for the time delay complex Chen system are shown in Figure 7.

## 5. Application and Simulation

In this section, we describe the simulation of the information transmission process for a real wireless body area network. We chose

- $h_{1}^{r}=35+5*rand\left(1,1\right)$ as the temperature, within the range of [35, 40] (°C);
- $h_{1}^{i}=70+130*rand\left(1,1\right)$ to stand for the high blood pressure value, within the range of [70, 200] (mmHg);
- $h_{2}^{r}=40+60*rand\left(1,1\right)$ to stand for the low blood pressure value, within the range of [40, 100] (mmHg);
- $h_{2}^{i}=60+40*rand\left(1,1\right)$ as the heart rate, within the range of [60, 100] (beats per minute, bpm); and
- $h_{3}=3.9+6.1*rand\left(1,1\right)$ as blood sugar, within the range of [3.9, 10] (mmol/L).

**Figure 8:**
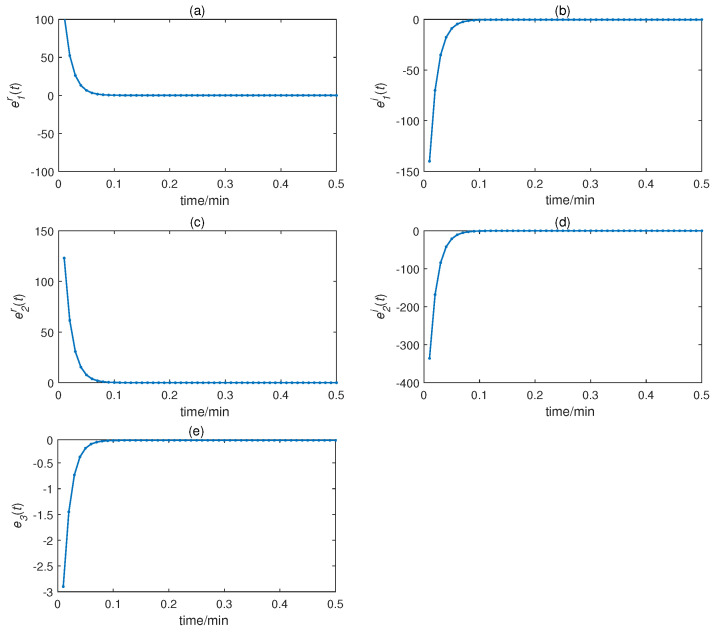
CMPS error for time delay complex Chen system: (**a**) $e_{1}^{r}\left(t\right)$, (**b**) $e_{1}^{i}\left(t\right)$, (**c**) $e_{2}^{r}\left(t\right)$, (**d**) $e_{2}^{i}\left(t\right)$, and (**e**) $e_{3}\left(t\right)$.

**Figure 9:**
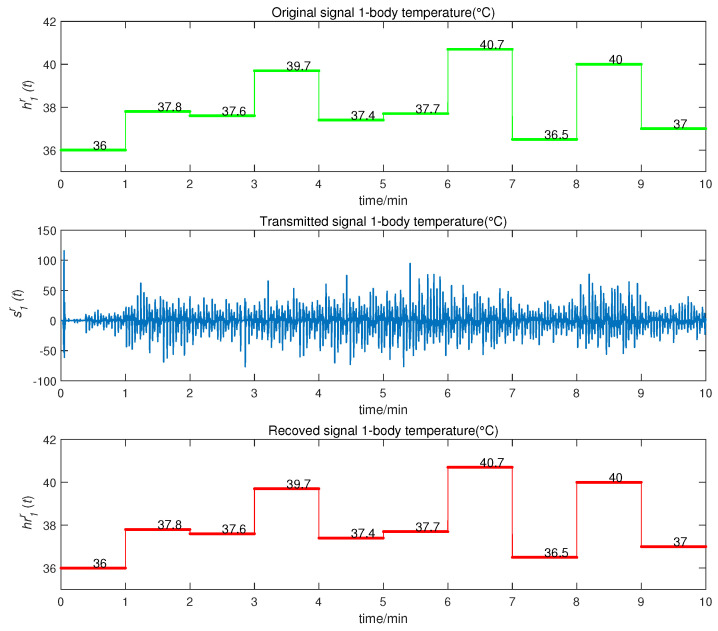
Temperature information transmission process.

**Figure 10:**
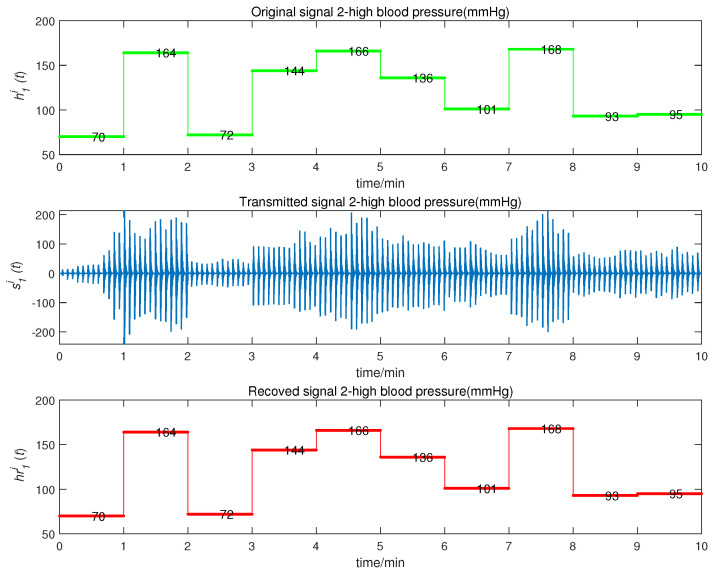
High blood pressure signal transmission process.

**Figure 11:**
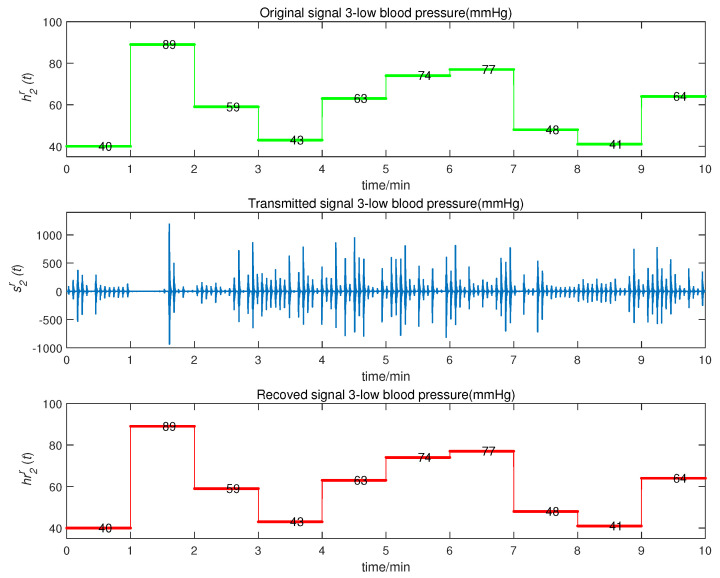
Low blood pressure signal transmission process.

**Figure 12:**
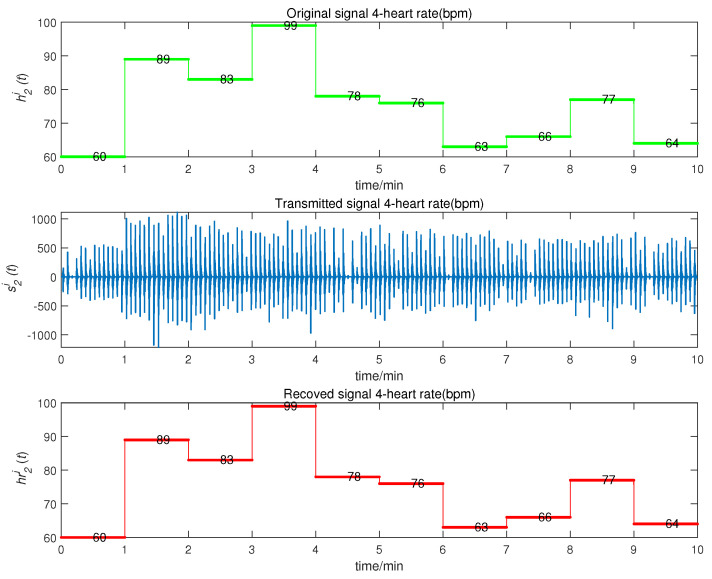
Heart rate signal transmission process.

**Figure 13:**
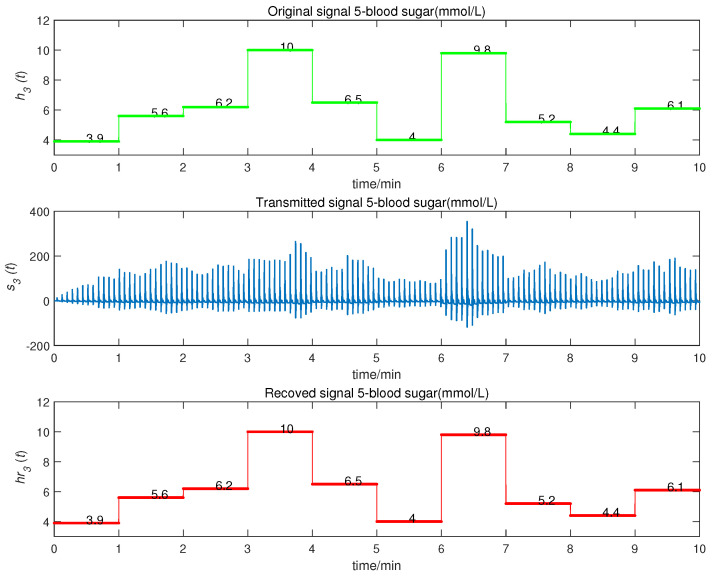
Blood sugar signal transmission process.

We obtained the CMPS error, as shown in Figure 8. The sender system collected data every minute, such that system errors were larger at the beginning of every minute, but quickly converged to zero. This indicates that CMPS takes place with complex scaling factors $h_{1},h_{2},h_{3}$. In reality, the physiological condition of the human body does not suddenly change and, so, the CMPS controller is able to maintain synchronization in one sampling period. The information transmission process is depicted in Figure 9, Figure 10, Figure 11, Figure 12 and Figure 13. The transmitted signal s(t) completely covered the information signal, while information signals h(t) were recovered with high precision. The above simulations demonstrate that the experimental results were in accordance with our theoretical analysis. The proposed communication system can quickly transmit information with high security.

## 6. Security Analysis

In this section, we analyze the security of the proposed CMPS communication scheme for WBAN.

### 6.1. Key Space Analysis

For the convenience of analysis, noise is not considered in the communication scheme. If a third party intercepts the signals of transmission channel such as s(t), as $s_{1}^{r}\left(t\right)=h_{1}^{r}g_{1}{\left(y\right)}^{r}-h_{1}^{i}g_{1}{\left(y\right)}^{i}$ or $s_{1}^{i}\left(t\right)=h_{1}^{r}g_{1}{\left(y\right)}^{i}+h_{1}^{i}g_{1}{\left(y\right)}^{r}$, the message signal *H* cannot be decrypted without the private keys such as the function g(y). In particular, when the amplitude of *H* is much smaller than that of g(y), s(t)≪g(y). It is impossible to decrypt the message signal *H* using only the signals of transmission channel. Therefore, for private keys g(y) include parameters, initial conditions and time lag τ, the key space of our algorithm is infinite.

### 6.2. Key Sensitivity Analysis

**Figure 14:**
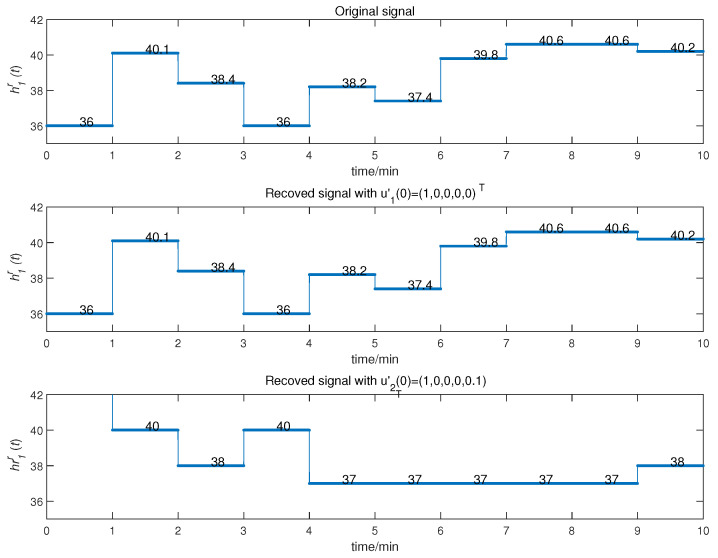
The CMPS process with $u_{1}^{\prime }\left(0\right)={\left(1,0,0,0,0\right)}^{T}$ and $u_{2}^{\prime }\left(0\right)={\left(1,0,0,0,0.1\right)}^{T}$.

In the proposed scheme, the most important private key is the system parameters and initial conditions. To analyze the sensitivity, the message signal *H* was transmitted with two close initial conditions $u_{1}^{\prime }\left(0\right)={\left(1,0,0,0,0\right)}^{T}$ in the senor node and $u_{2}^{\prime }\left(0\right)={\left(1,0,0,0,0.1\right)}^{T}$ at the sink node. The CMPS process is presented in Figure 14. The results demonstrate that the proposed algorithm has good sensitivity to the private key.

## 7. Conclusions

We first analyzed the characteristics of time delay complex Chen systems, outlining their randomness and unpredictability. Then, we proposed an innovative communication scheme based on CMPS for wireless body area networks and discussed its advantages. As the complex scale factor is more complicated than the real scale factor and the calculation of complex numbers is more complicated, the scheme can greatly increase the security of communication systems. Finally, we verified the effectiveness of the proposed communication system by conducting simulation experiments. The system could quickly and securely transmit information with strong robustness against noise. From the beginning, when Lorenz discovered and proposed chaos theory based on weather changes in nature, to the current chaos theory being applied in many fields, chaos theory has been constantly enriched and developed. The application of chaos communication theory in the field of WBANs is an important branch. The proposed chaotic communication scheme based on CMPS for WBANs only has its feasibility verified at the theoretical stage. It will be realized in the future, thus enhancing WBAN development.

## Figures and Tables

**Table 1:** Partial values of five Lyapunov indices of time delay complex Chen system.

Variable	${\mathrm{LE}}_{1}$	${\mathrm{LE}}_{2}$	${\mathrm{LE}}_{3}$	${\mathrm{LE}}_{4}$	${\mathrm{LE}}_{5}$	Symbol	Types
τ=0.5 s	2.2260	−0.0857	−0.7402	−0.5030	−1.1583	(+,0,−,−,−)	Chaos
τ=0.7 s	2.2837	0.7366	−0.0176	−0.5226	−1.4729	(+,+,0,−,−)	Hyperchaos
τ=1.5 s	0.4680	0.4676	−0.0298	−0.8371	−0.8366	(0,0,0,−,−)	Limit cycle

## Abbreviations

The following abbreviations are used in this manuscript:

CMPS	complex modified projection synchronization
WBAN	wireless body area network
PS	projection synchronization
MPS	modified projection synchronization
CS	complete synchronization
AS	anti synchronization
CCS	complex complete synchronization
BER	bit error rate
